# Genetic diversity and population structure analysis of a diverse panel of pea (*Pisum sativum*)

**DOI:** 10.3389/fgene.2024.1396888

**Published:** 2024-05-30

**Authors:** Haftom Brhane, Cecilia Hammenhag

**Affiliations:** Department of Plant Breeding, Swedish University of Agricultural Sciences, Lomma, Sweden

**Keywords:** DArT, genetic diversity, pea, population genetic structure, single-nucleotide polymorphism

## Abstract

Breeding resilient cultivars with increased tolerance to environmental stress and enhanced resistance to pests and diseases demands pre-breeding efforts that include understanding genetic diversity. This study aimed to evaluate the genetic diversity and population structure of 265 pea accessions. The diversity arrays technology (DArT) genotyping method was employed to identify single-nucleotide polymorphisms (SNPs) and silico markers. After stringent filtering, 6966 SNP and 8,454 silico markers were selected for diversity analysis. Genetic diversity was estimated by grouping accessions based on plant material type, geographic origin, growth habit, and seed color. Generally, diversity estimations obtained using SNPs were similar to those estimated using silico markers. The polymorphism information content (PIC) of the SNP markers ranged from 0.0 to 0.5, with a quarter of them displaying PIC values exceeding 0.4, making them highly informative. Analysis based on plant material type revealed narrow observed heterozygosity (Ho = 0.02–0.03) and expected heterozygosity (He = 0.26–0.31), with landrace accessions exhibiting the highest diversity. Geographic origin-based diversity analysis revealed Ho = 0.02–0.03 and He = 0.22 to 0.30, with European accessions showing the greatest diversity. Moreover, private alleles unique to landrace (4) and European (22) accessions were also identified, which merit further investigation for their potential association with desirable traits. The analysis of molecular variance revealed a highly significant genetic differentiation among accession groups classified by seed color, growth habit, plant material types, and geographic origin (*p* < 0.01). Principal coordinate analysis and neighbor-joining cluster analysis revealed weak clustering of accessions at different grouping levels. This study underscores the significance of genetic diversity in pea collections, offering valuable insights for targeted breeding and conservation efforts. By leveraging genomic data and exploring untapped genetic resources, pea breeding programs can be fortified to ensure sustainable plant protein production and address future challenges in agriculture.

## 1 Introduction

In the global north, pulses are currently transforming from primary use as animal feed to becoming an essential part of a plant-dominated human diet (Szczebyło et al., 2019). In Europe, over 80% of the cultivated faba beans (*Vicia faba*) and peas (*Pisum sativum*) are currently allocated for animal feed (Commission, 2018). Meanwhile, according to a European plant-based food and beverage market 2023–2030 report, the market for plant-based food products, in which pulses play a vital role, is rapidly expanding and projected to grow by approximately 11% annually until 2030. Some pulses have begun to move beyond traditional consumption as whole cooked seeds and are now increasingly used in novel applications such as flour and ingredients in meat analogs and various other food products (Ahmad et al., 2022; Dueholm et al., 2024). Additionally, ongoing climate change demands cultivars with enhanced abiotic resilience and improved resistance or tolerance to pests and diseases. This necessitates the development of improved cultivars with novel qualities and traits, many of which may not be present in current modern cultivars. Hence, there is a need, through pre-breeding efforts, to conduct comprehensive genetic screenings and evaluations to identify desirable traits within various wild and less improved populations.

Genetic diversity is critical for enhancing breeding programs. It provides the essential genetic resources needed for developing improved cultivars, ensuring resilience, high yield, and nutritional value, and adapting to changing environmental conditions and global food demands. Landraces and crop wild relatives play a crucial role in preserving genetic diversity in crops, harboring a wide array of unique traits and alleles that can be vital for future breeding programs (Dwivedi et al., 2016; Marone et al., 2021). However, characterizing landraces and wild populations poses a challenge due to their heterogeneous nature, as they display vast phenotypic variation and genetic complexity. This variability makes it difficult to define clear and distinct traits, requiring comprehensive genotypic and phenotypic evaluations to unravel their genetic composition and identify traits of interest.

Pea (*P. sativum* L.) is a diploid (*2n = 2x = 14*) legume that belongs to species of Fabaceae*,* subfamily Papillionaceae, and the tribe Vicieae with a genome size of about 4,500 Mb (Jain et al., 2014; Yang et al., 2022; Rispail et al., 2023). Despite the pea’s historical significance in genetics dating back to Mendel’s pioneering work, genomic resources have only recently undergone notable improvements due to the release of the whole-genome sequence Kreplak et al. (2019) and subsequent development of a reference genome and a pan-genome (Yang et al., 2022). These resources have provided valuable insights into the genetic makeup of the pea, facilitating more comprehensive genetic screenings and evaluations.

Pea diversity panels, composed of cultivars and less improved material, have previously been characterized using a wide range of markers such as amplified fragment length polymorphisms (AFLPs) and randomly amplified DNA polymorphisms (RADPs), followed by simple sequence repeats (SSRs), and a varying number of more recent single-nucleotide polymorphism (SNP) markers (Simioniuc et al., 2002; Burstin et al., 2015; Uhlarik et al., 2022). These studies have provided important knowledge and understanding of the existing genetic variation present in peas. However, despite including landraces and other diverse materials in the studies, only a limited number of these studies have taken the genetic heterogeneity of the landraces, gene bank accessions, and wild samples into account. As such, a large fraction of the genetic diversity that can be accessed within landraces and more diverse accessions has not been sufficiently investigated.

Plant breeding relies on selecting diverse germplasm with desirable traits and optimum seed sample size to develop new cultivars and conserve without disturbing the genetic integrity and variability (Crossa, 1989). This selection process involves understanding the genetic variations among the germplasm used in breeding. Therefore, utilizing genome-wide markers becomes crucial for uncovering the genetic diversity within the gene pool that can serve as tools for strategic conservation and plant breeding programs (Peterson et al., 2014). Diversity arrays technology sequencing (DArTseq) enables the discovery of genetic markers for high-throughput genotyping with little or no available genome sequence information (Kilian et al., 2012). The DArT markers are known for their efficiency and robustness and have been widely utilized in various crop plants, including peas, for genetic analyses, aiding in understanding genetic diversity (Wenzl et al., 2004; Robbana et al., 2019; Alemu et al., 2022).

The objectives of this study were to characterize genetic variation using DNA markers in a diverse collection of peas sampled from a broad geographical region and composed of different material types and characteristics. Along with a deeper exploration of the diversity within accessions known or expected to be more heterogeneous, such as landraces, wild material, and unspecified gene bank accessions, this study provides genetic resources and knowledge of the available germplasm as a contribution to the development of improved pea cultivars.

## 2 Materials and methods

### 2.1 Plant materials and their growth condition

**FIGURE 1:**
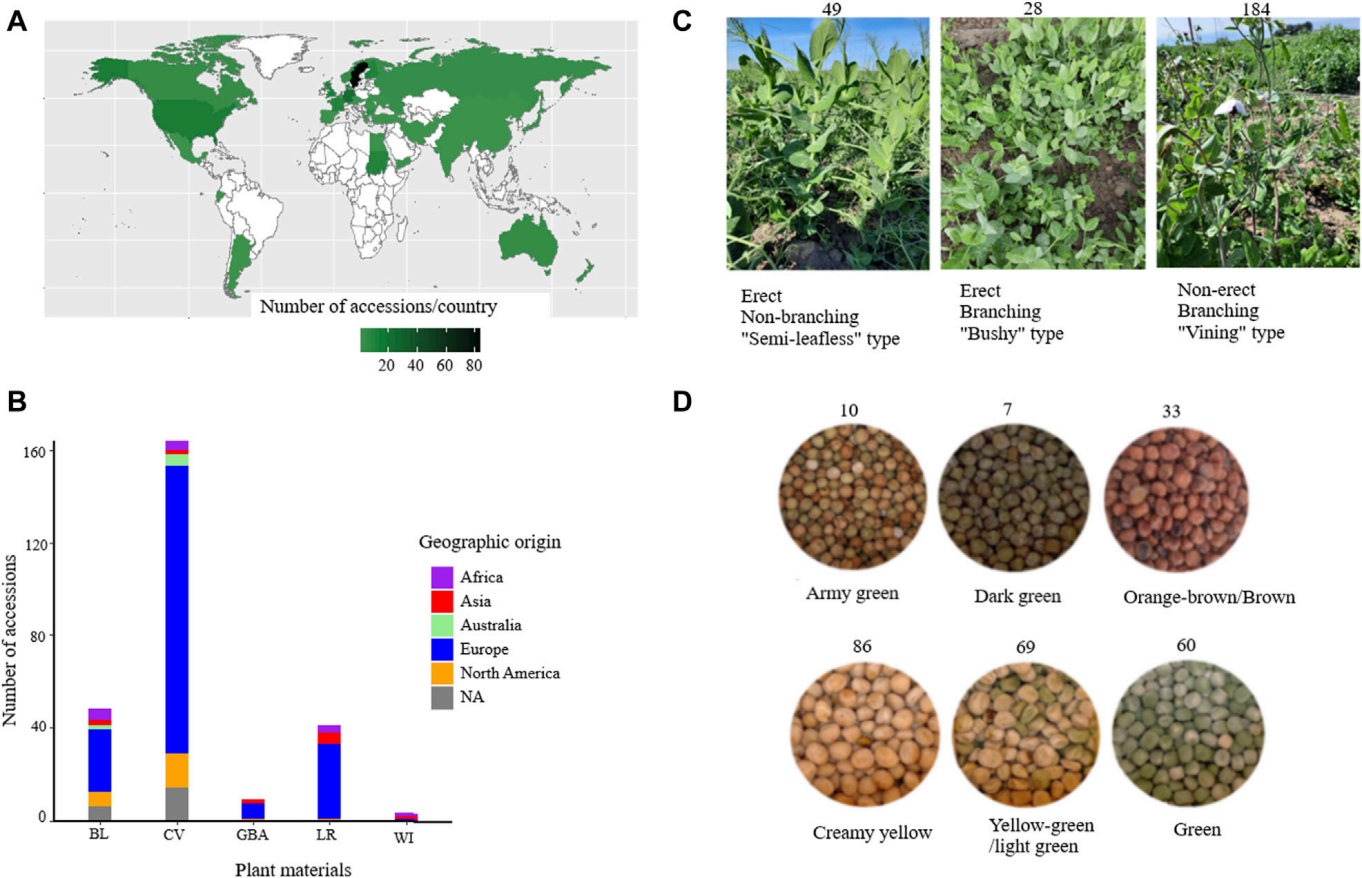
Overview of the accessions used in this study grouped based on their geographic origin **(A)**, plant material type **(B)**, growth habit **(C),** and seed color **(D)**. Abbreviations: BL = breeding lines, CV = improved cultivars, GBA = gene bank accessions, LR = landraces, and WI = wild materials.

In this study, a diverse set of plant materials comprising 265 accessions were sourced from global gene banks: International Center for Agricultural Research in the Dry Areas (ICARDA) (LBN002)), Nordgen (Nordic Genetic Resource Centre (SWE054)), ILRI (International Livestock Research Institute (ETH013)), National Institute for Agricultural Research, Food and Environment (INRAE (FRA043)), and Genebank of Leibniz Institute of Plant Genetics and Crop Plant Research (DEU146) and from breeders in Sweden and seed companies in Europe (Supplementary Table S1). The accessions were classified into five categories based on passport data: wild (WI), landrace (LR), breeding line (BL), cultivar (CV), and accessions with unknown material type were designated as gene bank accession (GBA). From here on, for the sake of simplicity, the accessions were named Ps, followed by the type of plant material (BL, CV, LR, GBA, and WI) and three digits. For instance, PsBL214 represents a breeding line accession, and PsCV68 represents an improved cultivar. A heat map was generated using the ggplot2 package in R to visualize the geographical distribution and plant material type of the accessions (R Core Team, 2013) (Figure 1A).

Determination of growth types of the accessions was performed during a field trial in southern Sweden (55.90_N, 13.09_E) in 2021. However, three accessions (PsCV301, PsCV302, and PsCV303) were acquired at a later stage and not planted in the field, and one (PsCV174) failed to produce viable plants for assessment, resulting in growth habit data being available for only 261 accessions. Forty seeds of each accession (except PsCV301, PsCV302, and PsCV303) were planted in 1-square-meter plots, and accessions with taller plants and vining growth habits were provided with metal trellises for support. Two characteristics were assessed based on visual observations of the overall plant architecture and support requirements in the field in order to categorize growth types: 1) Branching type vs. non-branching (semi-leafless), where branching-type plants exhibit lateral branches or secondary stems extending from the main stem, while non-branching plants exhibited limited or no lateral branches and were often characterized by a more upright and streamlined (less “bushy”) growth habit. 2) Erect vs. non-erect (requiring support), where erect-type plants were capable of standing upright independently without external support, and plants that required trellis support to prevent lodging (bending or falling over) were designated as having a non-erect growth type.

Seed color categorization was performed following the method outlined by Santos et al. (2019). Seeds from each accession from the same batch used for planting were visually classified into eight distinct color categories. However, due to challenges in distinguishing certain colors from one another for specific accessions, two color categories were merged into one (yellow-green and light green, and orange-brown and brown), resulting in a final classification of six color categories: creamy yellow (CY), yellow-green/light green (YG/LG), green (G), army green (AG), dark green (DG), and orange-brown/brown (OB/B).

### 2.2 Sampling, DNA extraction, and genotyping

Leaf tissue for DNA extraction was sampled from each accession grown in a glasshouse at the Swedish University of Agricultural Sciences, Alnarp, Sweden, for 21 days. The cultivation conditions involved the use of high-pressure sodium (HPS) lamps to sustain a 16-h light and 8-h dark photoperiod, and the temperature was maintained at 21°C during the light cycle and adjusted to 18°C during the dark period with a consistent relative humidity of 60%. For the 211 accessions classified as BL (48), CV (162), and LR (1), samples were collected from 3-week-old seedlings of each accession individually by punching ten 6-mm-diameter discs from the leaves of each accession. For the remaining 54 accessions, classified as either WI (3), LR (40), unspecified GBA (9), or CV (2), 10 individuals per accession were separately sampled (a total of 540 samples), with one disc from each individual. Afterward, the leaf tissue was freeze-dried for 48 h before being sent to Intertek ScanBi Diagnostics (Alnarp, Sweden) for DNA isolation using the sbeadexTM plant DNA extraction kit (Biosearch Technologies, Hoddesdon, United Kingdom), followed by genome complexity reduction-based sequencing using Msel restriction enzyme at DArTseq (Diversity Array Technologies, Canberra, Australia), with a sequencing depth of 800,000 counts/sample. The generated sequence reads were then processed using proprietary DArT analytical pipelines.

### 2.3 Sequence read alignment, SNP discovery, and filtering

The generated raw reads were mapped to the recently released reference genome of *P. sativum* cultivar ZW6 (Yang et al., 2022). The mapping rate of the DArT reads to the reference genome was 99%. SNP discovery was performed using DArTsoft14 platform integrated in KDCompute plug-in system v.1.5.2 (https://kdcompute.seqart.net/kdcompute/login) (Diversity Arrays Technology, 2017). DArT-SNP and DArT-silico markers were scored in a binary fashion (1/0), yielding 25,708 and 14,830 raw markers, respectively. DArT-silico markers were scored in a binary fashion, representing genetically “dominant” markers, with “1” = Presence and “0” = Absence of a restriction fragment with the marker sequence in the genomic representation of the sample. “-” represents calls with non-zero counts but too low counts to score confidently as “1” (often representing heterozygotes). DArT-SNP markers were scored in a binary fashion (“1” = Presence and “0” = Absence), and heterozygotes were therefore scored as 1/1 (presence for both alleles/both rows). For the sake of simplicity, the DArT-SNP and DArT-silico markers will be referred to as “SNP” and “silico,” respectively. Highly informative markers were selected for diversity analysis after removing monomorphic, lower call rates (below 70%) and lower minor allele frequencies (MAF) below 0.05. Markers were not filtered by MAF to estimate the minimum seed sample size required to capture 95% of alleles within an accession with a 95% certainty to include the rare alleles, as described in Crossa (1989). Finally, the selected markers were imputed using the Fast Inbred Line Library ImputatioN (FILLIN) model (Swarts et al., 2014), which is integrated into TASSEL v.5 software and used for downstream analysis (Bradbury et al., 2007). To enrich the genomic information of the crop, the quality-trimmed sequence reads were deposited in a sequence reads archive (SRA) under the Bioproject number PRJNA1071600.

### 2.4 Data analysis

For a better understanding of the pattern of genetic diversity, the accessions were analyzed by grouping them according to their genetic material type, geographic origin, growth habit, and seed color. The genetic diversity of the 265 accessions was assessed using the selected highly informative markers by employing different genomic analysis software. The informativeness of the markers was evaluated after calculating polymorphism information content (PIC) (Botstein et al., 1980). The PIC value of each marker was estimated using PowerMarker v.3.25 (Liu and Muse, 2005). GeneAlEx v. 6.51b2 was used to estimate the number of alleles (Na), effective number of alleles (Ne), observed heterozygosity (Ho), expected heterozygosity (He), Shannon’s information index (I), gene diversity (h), and percentage of polymorphic loci (PPL) of the markers across the accessions (Peakall and Smouse, 2006).

Stringently filtered SNP markers were used to conduct genome-wide linkage disequilibrium (LD) analysis using Trait Analysis by Association, Evolution, and Linkage (TASSEL v. 5) with the default settings in a 50-sliding-window size. Pairwise squared allele-frequency correlations (r^2^) between SNP markers from the DArTseq were generated, and the r^2^ values were then plotted against the physical distance between the SNP loci to estimate the extent of LD between pairs of loci using R software.

The minimum seed sample size required to capture 95% of alleles within an accession with a 95% certainty was estimated in the R program following the Crossa (1989) methodology. Considering the rarest bi-allelic locus (SNP), two alleles, *B*1 and *B*2, with frequencies of *p*1 and *p*2, so that (*p*1 + *p*2 = 1), the two possible outcomes will be:

K1=B1 is not represented in the sample of n gametes.

K2=B2 is not represented in the sample of n gametes.

Thus, the probability of getting at least one copy of each *B*1 and *B*2 will be $P\left(K_{1}^{c}\cap K_{2}^{c}\right)$

$$P\left(K_{1}^{c}\cap K_{2}^{c}\right)=1-{\left(1-p1\right)}^{n}-{\left(1-p2\right)}^{n}.$$

The analysis of molecular variance (AMOVA) of the accessions based on different grouping was performed using Arlequine v. 3.5.2.2 (Excoffier and Lischer, 2010). The average number of Nei’s genetic distance (Nei, 1972) based on pairwise differences within and among the populations was also estimated using Arlequine. Principal coordinate analysis (PCoA) was used to visualize the genetic differentiation among the 265 pea accessions by grouping the genotypes at different levels, such as plant material, geographic origin, growth habit, seed color, and population genetics, using GeneAlEx v. 6.51b2 (Peakall and Smouse, 2006). Neighbor-joining (NJ) cluster analysis was generated based on Nei’s genetic distance among the populations using MEGA-7 software (Kumar et al., 2016). Furthermore, the genetic structure of the 265 pea accessions was evaluated using STRUCTURE 2.3.4 software (Pritchard et al., 2000). STRUCTURE analysis was run ten times with K = 1 to K = 10 using the admixture model with a burn-in period length of 100,000 and a Markov chain Monte Carlo of 100,000 replications.

## 3 Results

### 3.1 The germplasm collections

For this study, a total of 265 pea accessions were originally collected from different parts of the world, such as Africa (13), Asia (12), Australia (7), Europe (190), and North America (23), while 20 accessions had an unknown origin (NA) (Figure 1A). The collection panel also comprised breeding lines (BL = 48), improved cultivars (CV = 164), landraces (LR = 41), gene bank accessions (GBA = 9), and wild materials (WI = 3) (Figure 1B). The accessions represented different growth habits where 184 were classified as non-erect and branching type (NE and B), 28 were erect and branching (E and B), 49 were erect and non-branching (E and NB), and four were not identified types (NA) (Figure 1C). The accessions were categorized into six seed color groups, with the following distribution: creamy yellow (86), yellow-green/light green (69), green (60), army green (10), dark green (7), and orange-brown/brown (33) (Figure 1D).

### 3.2 Characteristics and distribution of the markers

The alignment of the raw reads from the 265 pea accessions to the reference genome resulted in the discovery of 25,708 SNPs and 14,830 silico markers. The markers were then filtered using DArT-specific criteria (minimum call rate above 70%, minimum allele frequency (MAF) ≥ 0.05, and minimum PIC value ≥ 0.05). Based on this, 6,966 SNPs and 8,458 silico markers were selected and used to estimate the genetic diversity of the accessions. Among the selected SNP markers, 1,350 had a call rate of between 0.70 and 0.80, and more than 50% of them were present in 90% of the accessions. In addition, the allele frequency distribution of the markers indicated that 2,897 ranged between 0.05 and 0.5 and 4,069 ranged between 0.50 and 1.0. In total, the PIC value of the markers ranged between 0.0 and 0.5, with most (2,616) being between 0.40 and 0.50 (Table 1). The estimated characteristics of the SNP markers were broadly similar to those of silico markers, except the silico markers were large in number. In the silico marker set, there were no markers below the 0.80 call rate in the genotypes, all being either 1 or 0. Most markers (6,350) were between 0.90 and 1.00. Distribution of the allele frequencies ranged from 0.05 to 1.00, with most distributions being between 0.05 and 0.5. The PIC values ranged from 0.05 to 0.50, and only 46 fell between 0.00 and 0.10 (Table 1).

**TABLE 1:** Overview of the distribution of marker quality parameters in the form of call rate, minor allele frequency (MAF), and polymorphic information content (PIC) of the 6966 SNP and 8,458 silico markers.

	Range	SNP	Silico
Call rate	0.70–0.80	1,350	0
	0.80–0.90	2,108	2,128
	0.90–1.00	3,508	6,350
MAF	0.05–0.50	2,897	5,329
	0.50–1.00	4,069	3,129
PIC	0.00–0.10	52	46
	0.10–0.25	2,345	2,614
	0.25–0.40	1953	2,588
	0.40–0.50	2,616	3,210

**FIGURE 2:**
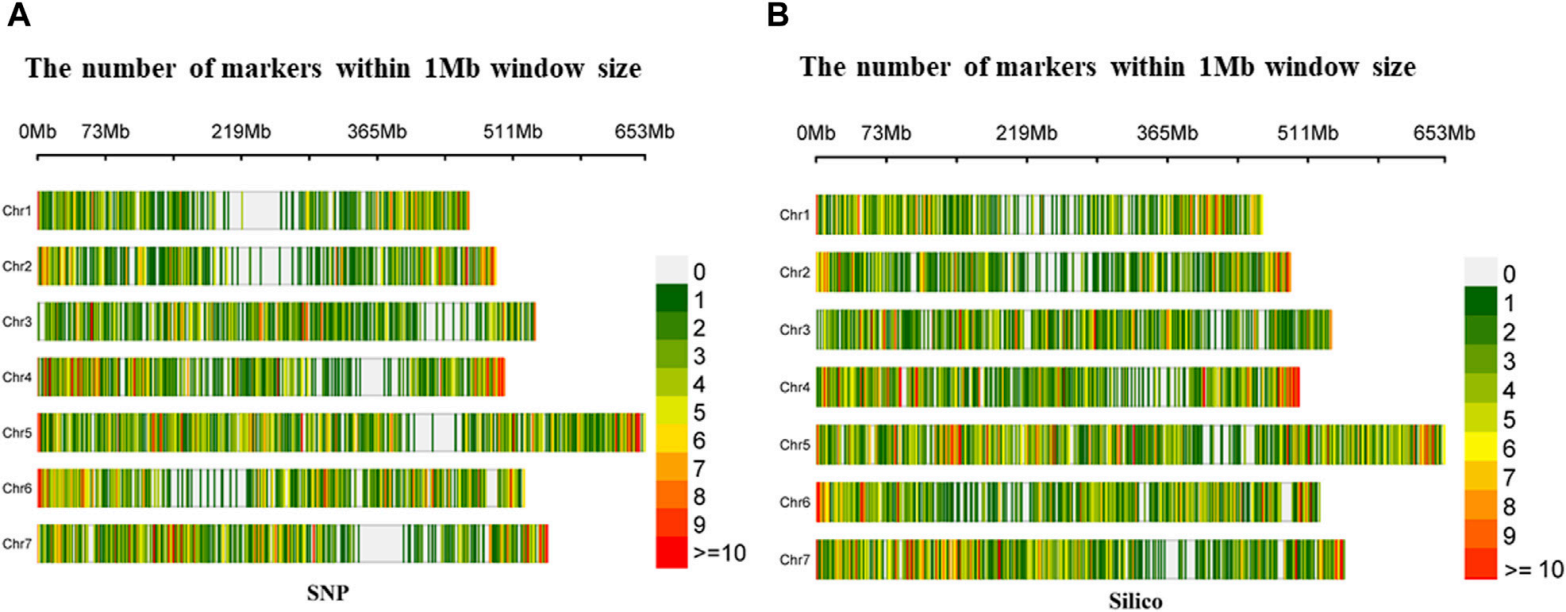
Density distribution of **(A)** diversity array technology-derived SNP and **(B)** diversity array technology-derived silico markers in a 1 Mb window across the seven pea chromosomes.

The density distribution of the selected markers from the SNP and silico approaches had uniform coverage across the seven chromosomes with minimal gaps in the former. In the SNP markers, the highest number of markers (1,422) was found on Chromosome 5 (Chr5), while the smallest number (734) was found on Chromosome 1 (Chr1). Furthermore, from the silico marker, the largest number of markers (1,681) was found on Chromosome 5 (Chr5) and the smallest number (909) on Chromosome 2 (Chr2) (Figure 2).

**FIGURE 3:**
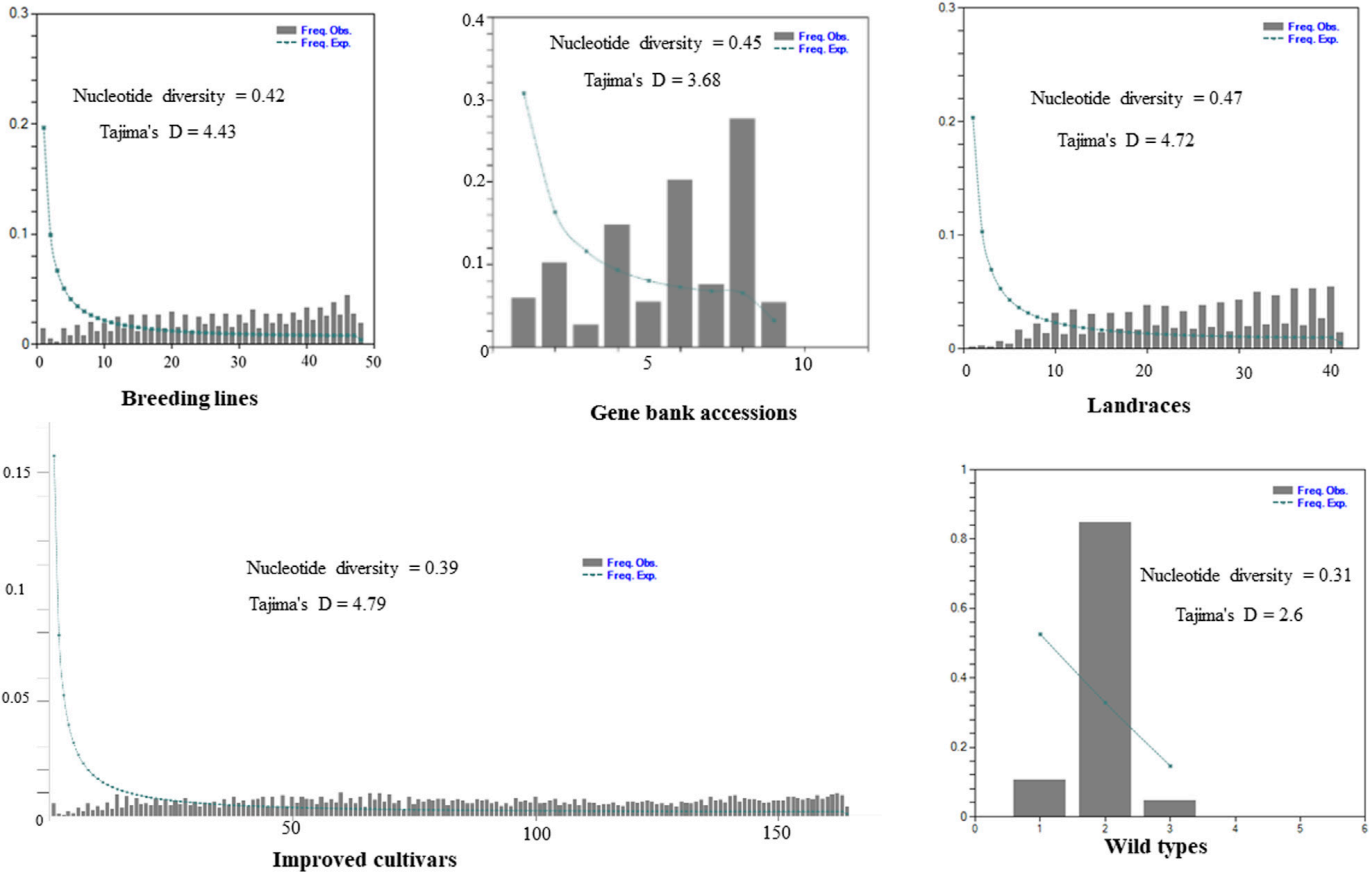
Graph depicting minor allele frequency (MAF) based site-frequency spectrum, nucleotide diversity, and Tajima’s D test across the five distinct plant material types of pea populations evaluated using 6966 SNP loci.

The site-frequency spectrum revealed that the MAF distribution of the SNP loci varied substantially among the five distinct types of plant material-based pea populations. The MAF of the wild-type accessions varied from 0.04 to 0.84, with PsWI18 showing the highest observed frequency compared to the expected frequency (0.84). The distribution of MAF in the gene bank accessions also ranged from 0.01 to 0.19, whereas the improved cultivars ranged from 0.0007 to 0.009. This indicates that the MAF contributes to the level of genetic variation among the different populations to a different degree. Moreover, the gene bank accessions and landrace plant material had a higher nucleotide diversity, 0.45 and 0.47, respectively. The nucleotide diversity of the improved cultivars was 0.39, and it was 0.31 for the wild materials (Figure 3).

### 3.3 Genetic diversity across populations

Genetic diversity estimations were made based on the two marker types to understand the genetic variation and relationships within and among the collection of accessions. Overall, the genetic diversity estimations obtained for SNP markers were broadly similar to those estimated using silico markers. The filtered markers were used to estimate the level of diversity in the populations grouped based on plant material type, and landrace accessions were found to be more diverse (Ne = 1.52, I = 0.48, He = 0.31). Improved cultivars (Ne = 1.44, I = 0.41, He = 0.27, and number of private alleles (NPA) = 0) and wild materials (Ne = 1.47, I = 0.37, He = 0.26, and NPA = 0) were found to be relatively less diverse populations. The percentage of polymorphic loci (PPL) was higher (100%) in landrace accessions than in other plant material types (Table 2). Among the plant material types, private alleles were found only in landrace accessions (NPA = 4) (Supplementary Table S2).

**TABLE 2:** Average values of genetic diversity parameters such as number of accessions (N), number of alleles (Na), effective number of alleles (Ne), Shannon index (I), gene diversity (h), observed heterozygosity (Ho), expected heterozygosity (He), number of private alleles (NPA), and percentage of polymorphic loci (PPL).

	Pop	N	Na	Ne	I	Ho	He	NPA	PPL
**SNP**	Breeding lines^a^	48	1.96	1.48	0.44	0.02	0.29	0	96.4
	Improved cultivars^a^	164	1.98	1.44	0.41	0.02	0.27	0	98.4
	Landraces^a^	41	2	1.52	0.48	0.02	0.31	4	100
	Gene bank accessions^a^	9	1.89	1.51	0.45	0.03	0.3	0	96.7
	Wild materials^a^	3	1.6	1.47	0.37	0.03	0.26	0	82.4
	Total	265	1.89	1.48	0.43	0.02	0.29	4	94.8
	Africa^b^	13	1.86	1.48	0.43	0.02	0.28	0	79
	Asia^b^	12	1.86	1.49	0.44	0.03	0.29	0	85
	Australia^b^	7	1.64	1.38	0.34	0.02	0.22	0	62
	Europe^b^	190	2.00	1.48	0.45	0.02	0.30	22	100
	North America^b^	23	1.91	1.46	0.43	0.02	0.28	0	88
	Unknown materials^b^	20	1.89	1.44	0.41	0.02	0.27	0	99
	Total	265	1.86	1.45	0.42	0.02	0.27	22	85
	**Pop**	**N**	**Na**	**Ne**	**I**	**h**	**NPA**	**PPL**	
**Silico**	Breeding lines^a^	48	1.95	1.47	0.44	0.29	0	94.8	
	Improved cultivars^a^	164	1.97	1.45	0.42	0.27	0	97.2	
	Landraces^a^	41	1.95	1.49	0.45	0.29	0	94.8	
	Gene bank accessions^a^	9	2	1.52	0.48	0.32	0	99.8	
	Wild materials^a^	3	1.8	1.51	0.43	0.29	0	79.8	
	Total	265	1.93	1.49	0.44	0.29	0	93.2	
	Africa^b^	13	1.7	1.47	0.41	0.28	0	81.6	
	Asia^b^	12	1.76	1.51	0.45	0.3	0	85.5	
	Australia^b^	7	1.4	1.39	0.34	0.23	0	61.8	
	Europe^b^	190	2	1.51	0.47	0.31	0	99.8	
	North America^b^	23	1.82	1.5	0.45	0.29	0	89.5	
	Unknown materials^b^	20	1.96	1.45	0.42	0.28	0	97.3	
	Total	265	1.77	1.47	0.42	0.28	0	85.9	

Accessions grouped based on

^a^ Plant material types and

^b^ Geographic origin.

The same markers were used to estimate the level of genetic diversity of the accessions grouped based on their geographic origin. This revealed accessions collected from Europe had relatively higher genetic diversity parameters (number of alleles (Na = 2.00), number of effective alleles (Ne = 1.48), Shannon index (I = 0.45), expected heterozygosity (He = 0.30), and NPA = 22). Accessions collected from Africa (I = 0.43 and He = 0.28), Asia (I = 0.44 and He = 0.29), and North America (I = 0.43 and He = 0.28) were represented by a similar number of accessions and showed a relatively similar pattern of genetic diversity. In addition, the accessions grouped as “GBA” (unknown geographic origin) were also in the range of diverse groups (Table 2).

Performing the same analysis using the silico markers also confirmed that the accessions collected from Europe were found to be relatively slightly diverse materials (I = 0.47, h = 0.31). The accessions collected from Africa, Asia, and North America showed similar patterns of genetic diversity. In addition, landrace accessions were relatively heterogeneous materials (I = 0.48, and h = 0.32) compared to other types of plant materials. The PPL of the silico markers was also relatively higher (99.8%) in accessions collected from Europe when the accessions were grouped based on geographic origin and 100% in landrace accessions when grouped based on the plant material types (Table 2). The minimum seed sample size required to preserve the genetic variability existing in each pea landrace, gene bank accessions, and wild materials with 95% of the alleles with an expected probability of 95% was estimated using the DArTseq-SNPs. Seed sample size ranged from 256 to 668 (Supplementary Table S3). The results of the seed sample size required to preserve the rare alleles from each accession at 90%, 95%, and 99% certainty are given in Supplementary Table S3.

### 3.4 Genetic variation within and among different groups of accessions

Analysis of molecular variance (AMOVA) revealed significant genetic variations within and among different groups of the pea accessions. As the accessions denoted as landraces and the unspecified gene bank accessions were expected to be composed of several genotypes, ten individuals were sampled from each of these accessions. AMOVA on these 54 accessions, including the 10 individuals in each accession, revealed higher within-population variation (90%) than among-population variation (10%). AMOVA was also estimated on these accessions by including one randomly selected individual per accession to observe the difference in diversity due to sampling. The analysis showed substantial differences among- (3%) and within-population (97%) genetic variation.

AMOVA estimated the 265 accessions by including one randomly selected individual (out of ten) from the accessions denoted as landraces, gene bank accessions, or wild revealed higher within-population variation (95%) than among-population variation (5%). Similarly, higher within-population variation was also obtained when the accessions were grouped based on geographic origin (95.8%), growth habit (93%), and seed color (93%) (Table 3). Notably, the genetic variation among and within the defined groups was highly significant (*p* < 0.001). The genetic differentiation of the pea populations (FI) was low, that is, 0.04 for geographic-based groups, followed by 0.05 for the five plant material-based groups, 0.07 for the growth habit groups, and 0.07 for seed color-based groups. In contrast, there was high genetic differentiation among the accessions (0.10), which were represented by 10 individuals that showed distinct genetic profiles for each accession (Table 3).

**TABLE 3:** Analysis of molecular variance (AMOVA) of the 265 pea accessions at different levels of grouping, such as sample representation, type of plant material, geographic origin, growth habit, and seed color, using the 6966 SNP markers.

Source of variation	DF^Z^	SS	Est. var	PV (%)	FI	*p*-value
Populations represented by one individual						
Among accessions	3	16482.4	142.9	3.0	0.03	*p* < 0.001
Within accessions	50	220800.6	4,416.0	97		
Populations represented by 10 individuals						
Among populations	3	120529.4	480.0	10	0.10	*p* < 0.001
Within populations	535	2250024.8	4,205.6	90		
Plant material type-based grouping						
Among populations	4	43540.9	186.2	5.0	0.05	*p* < 0.001
Within populations	260	994778.3	3,826.0	95		
Geographic origin-based grouping						
Among populations	5	15887.1	98.7	4.22	0.04	*p* < 0.001
Within populations	259	522547.9	977	95.8		
Growth habit-based grouping						
Among populations	3	45580.0	273.0	7.0	0.07	*p* < 0.001
Within populations	261	992738.9	3,803.6	93		
Seed color-based grouping						
Among populations	5	87017.4	77.8	7.0	0.07	*p* < 0.001
Within populations	259	951301.4	959.8	93		

DF^Z^.

Degrees of freedom; SS, sum of squares; Est. var, estimated variance; PV, percentage of variation; FI, fixation index.

### 3.5 Pairwise genetic distance, principal coordinate analyses, and cluster

The pairwise Nei’s standard genetic distance calculated across different groups revealed a spectrum of genetic relationships among populations. Across various types of genetic materials, the observed genetic distances ranged from 0.01 to 0.13. It is worth noting that the shortest genetic distance (0.01) was observed between breeding lines and improved cultivars, with a slightly higher distance (0.02) between breeding lines and landraces. On the other hand, the widest genetic distances were observed between wild materials and improved cultivars (0.13), followed by wild materials and breeding lines (0.11) and wild materials and landraces (0.11) (Table 4A).

**TABLE 4:** Average number of pairwise genetic differences among the different pea populations categorized as types of plant materials (A), geographic origin (B), growth habit (C), and seed color (D). Abbreviations: *NE and B = Non-erect and branching, E and B = erect and branching, E and NB = erect and non-branching, and NA = not identified.*

A) Plant materials	Breeding lines	Improved cultivars	Gene bank accession	Landraces	Wild materials	
Breeding lines	0.00					
Improved cultivars	0.01	0.00				
Gene bank accessions	0.04	0.05	0.00			
Landraces	0.03	0.03	0.04	0.00		
Wild materials	0.12	0.13	0.11	0.11	0.00	
**B) Geographic origin**	Africa	Asia	Australia	Europe	NA	North America
Africa	0.00					
Asia	0.06	0.00				
Australia	0.09	0.06	0.00			
Europe	0.05	0.03	0.04	0.00		
NA	0.06	0.04	0.04	0.02	0.00	
North America	0.06	0.03	0.04	0.01	0.02	0.00
**C) Growth habit**	NE and B	E and B	E and NB	NA		
NE and B	0.00					
E and B	0.02	0.00				
E and NB	0.04	0.04	0.00			
NA	0.09	0.11	0.09	0.00		
**D) Seed color**	Creamy yellow	Yellow-green/light green	Green	Army green	Dark green	Orange-brown/Brown
Creamy yellow	0.00					
Yellow-green/light green	0.03	0.00				
Green	0.02	0.01	0.00			
Army green	0.04	0.06	0.05	0.00		
Dark green	0.10	0.11	0.10	0.07	0.00	
Orange-brown/brown	0.04	0.06	0.06	0.03	0.06	0.00

The genetic distance of the accessions grouped based on their geographic origin revealed genetically close populations, such as Europe and North America (0.01), followed by between accessions from Europe vs. unknown materials (0.02). In contrast, a notably higher genetic distance was observed between accessions originating from Africa and Australia (0.09). Interestingly, the accessions with unknown geographic origin showed high genetic similarity with European accessions (Table 4B).

Furthermore, pairwise genetic distance between the growth habit groups ranged between 0.01 and 0.10. The shortest genetic distance, 0.02, was obtained between erect and branching vs. non-erect and branching. Conversely, the highest genetic distance, 0.11, was observed between the accessions not identified vs. erect and branching (Table 4C).

In addition, pairwise mean genetic distance of the seed color-based grouping ranged between 0.01 and 0.11. The shortest genetic distance, 0.01, was found to be between accessions with green and yellow-green/light green, followed by 0.02 for green vs. creamy yellow accessions. In contrast, the highest genetic distance, 0.11, was found between yellow green/light green vs. dark green accessions, followed by 0.10 between dark green vs. creamy yellow accessions (Table 4D).

**FIGURE 4:**
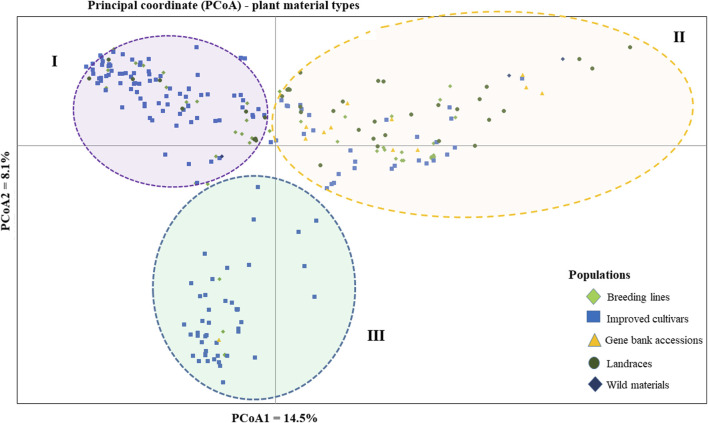
Bi-plot of principal coordinate analysis (PCoA) depicting the genetic relationship of the 265 pea accessions grouped according to their genetic material type generated using 6966 SNP markers. Symbols sharing the same shape and color indicate accessions of the same population.

The PCoA was conducted to visualize the genetic diversity among 265 pea accessions. The findings revealed that the first and second coordinates accounted for 22.6% of the total variation. Although the accessions showed weak clustering based on their genetic material, they predominantly grouped into three clusters (I, II, and III). Notably, the first coordinate, which contributed 14.5% of the total variation, played a significant role in this clustering. Cluster-I comprised accessions of breeding lines, improved cultivars, and landrace materials, with genotypes PsCV68 and PsCV71 positioned distantly in this cluster. Cluster-II showed higher heterogeneity, composed of a mixture of accessions of all different material types. Cluster-III was characterized by a greater degree of homogeneity and was composed of improved cultivars, with only three breeding lines and one gene bank accession interspersed within the cluster (Figure 4).

**FIGURE 5:**
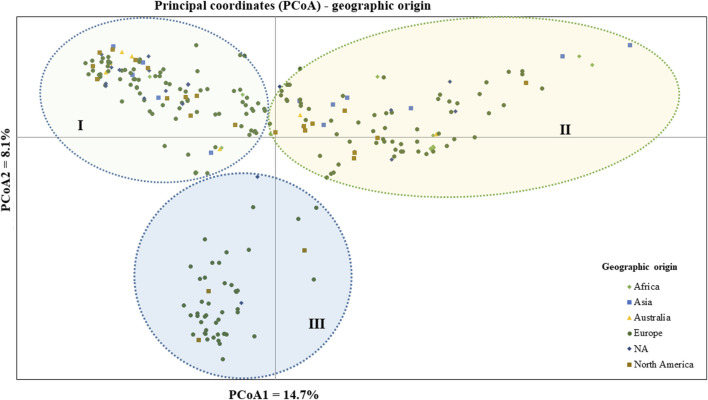
Bi-plot of principal coordinate analysis (PCoA) depicting the genetic relationship of the 265 pea accessions grouped according to their geographic origin generated using 6966 SNP markers. Symbols sharing the same shape and color indicate accessions of the same population.

The geographic origin of the genotype-based PCoA revealed that the first and second coordinates together explained a total of 22.7% total variation. Three (I, II, and III) heterogeneous clusters were obtained, indicating a lack of clustering based on their geographic origin. Cluster-I was composed of heterogeneous accessions representing different geographic origins. Notably, accessions PsCV189, PsCV68, PsCV69, PsCV106, PsCV184, PsCV203, PsWI18, and PsBL278 were distantly placed from the other genotypes within this cluster. Several of these accessions are short-statured garden pea cultivars. Similarly, Cluster-II was composed of heterogeneous accessions representing different geographic origins, with genotypes such as PsLR214, PsLR209, PsWI21, and PsCV294, all non-European material, being placed distantly within this cluster. In contrast, Cluster-III comprised homogenous accessions representing collections from Europe and without known origin, with PsCV205, PsCV168, PsCV122, PsCV210, PsCV134, and PsCV133 genotypes placed distantly in this cluster (Figure 5).

**FIGURE 6:**
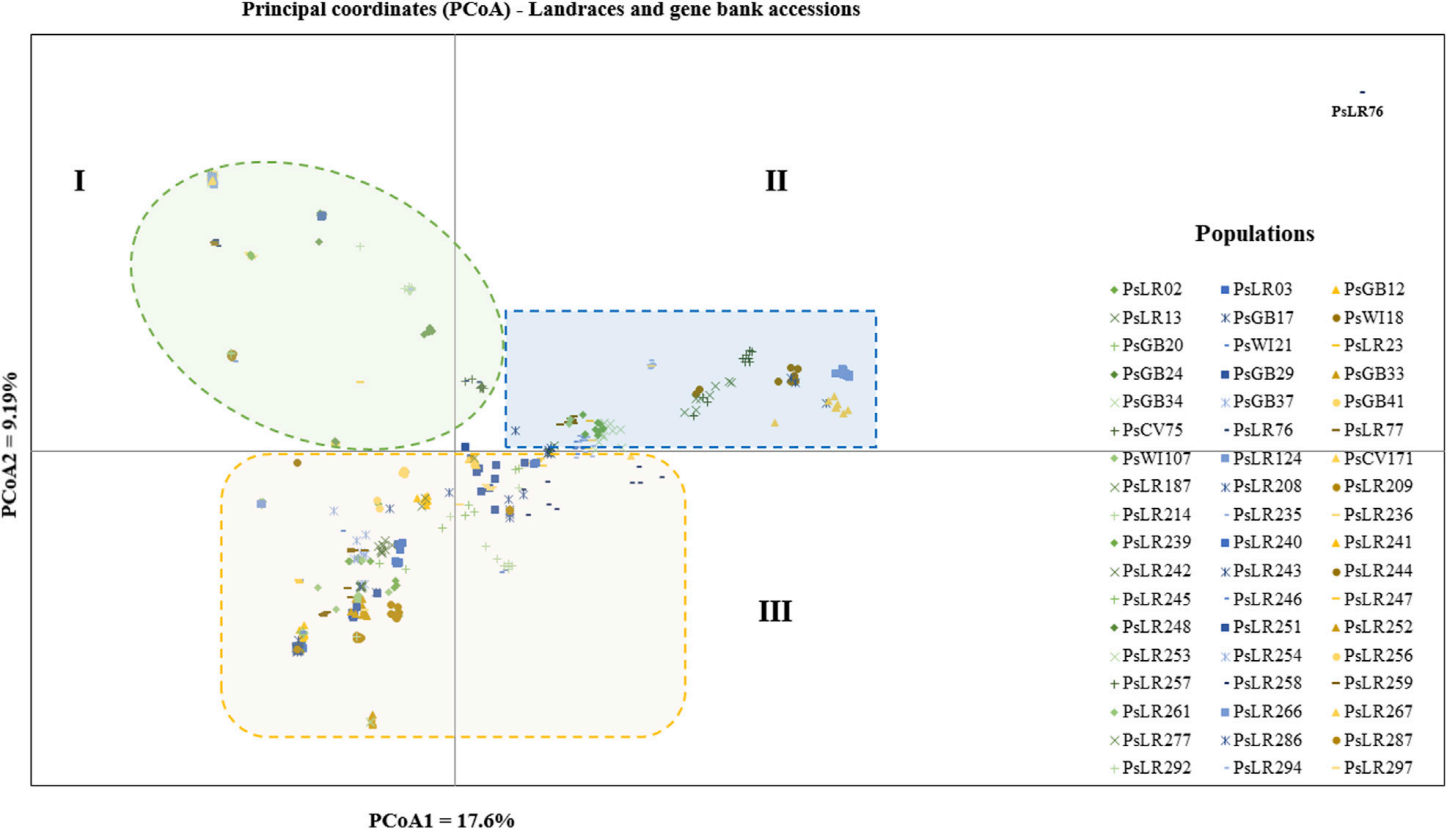
Bi-plot of principal coordinate analysis (PCoA) depicting the genetic relationship among the 54 pea populations generated using 6966 SNP markers. Each population is represented by 10 individuals. Symbols sharing the same shape and color indicate accessions of the same type of plant material.

**FIGURE 7:**
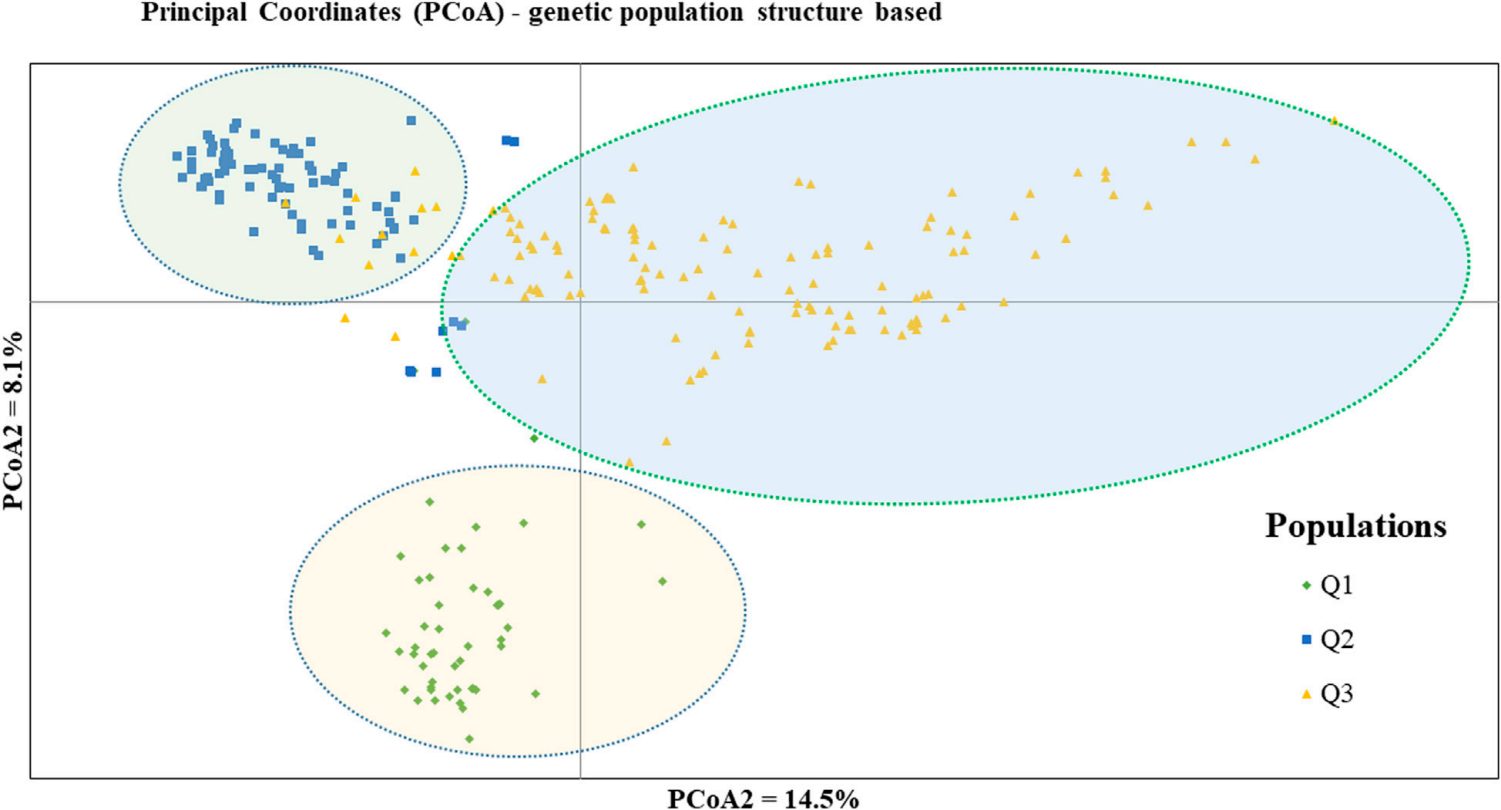
Bi-plot of principal coordinate analysis (PCoA) depicting the genetic relationship of the 265 pea accessions grouped according to their population genetic structure generated using 6966 SNP markers. Symbols of the same shape and color indicate accessions of the same population genetic group.

**FIGURE 8:**
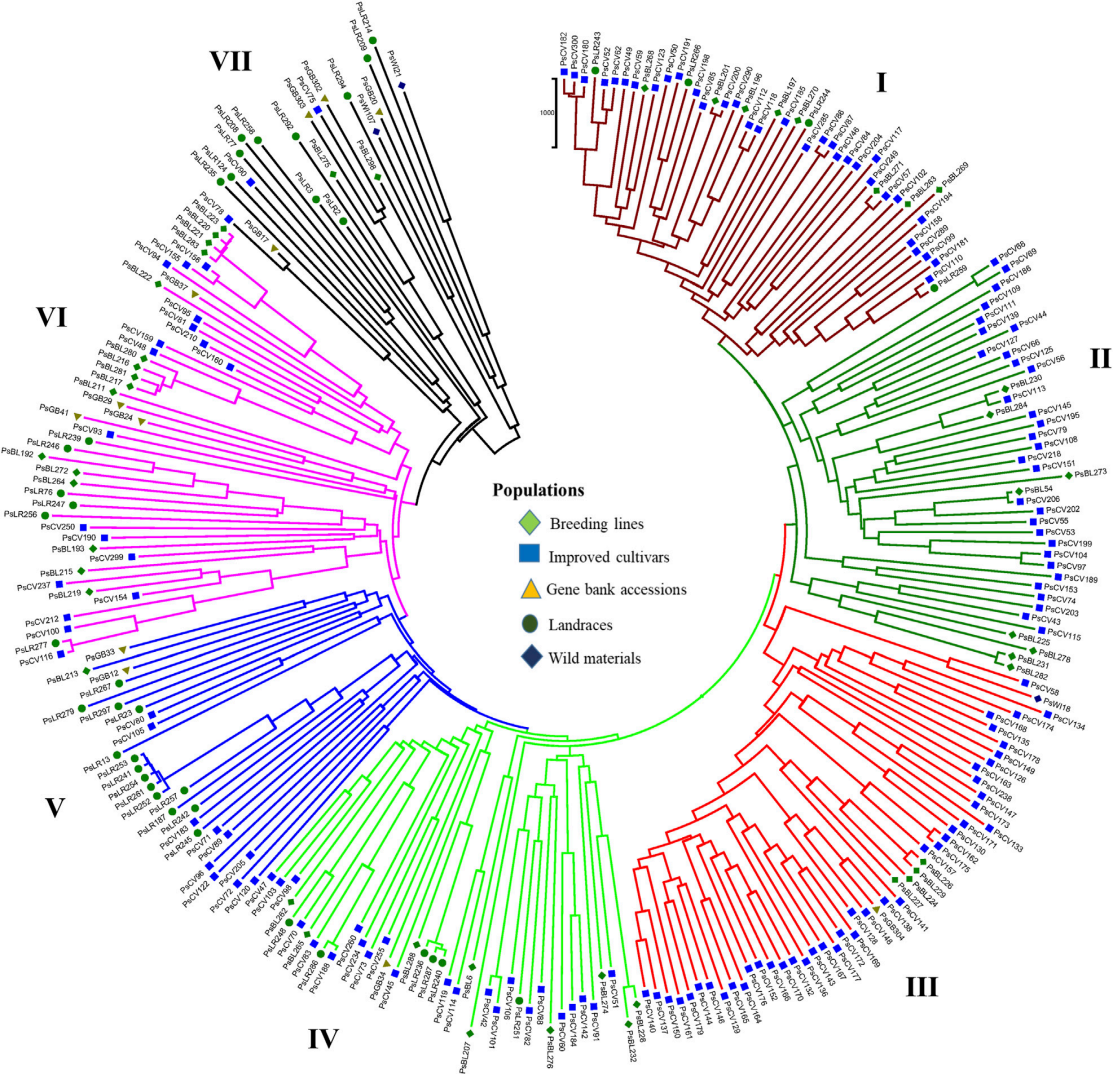
Nei’s standard genetic distance neighbor-joining (NJ) clustering analysis of 265 pea accessions grouped according to plant material type was generated using 6966 SNP markers. Accessions sharing symbols with the same shape and color belong to the same plant material type.

PCoA was also employed to visualize the genetic differentiation among the 54 pea genotypes, which were represented by 10 individuals each. This resulted in a total genetic variation of 26.7% among the genotypes, of which the first coordinate alone contributed 17.6%. The bi-plot broadly showed three (I, II, and III) clusters of the 54 genotypes. Notably, with few exceptions, almost all the 10 individual plants from each genotype were closely grouped together, indicating a high level of genetic identity among the individuals sampled from the same genotype Figure 6). Compared to the genotypes in Cluster-II and Cluster-III, Cluster-I genotypes exhibited greater genetic distance from each other. An individual outlier from accession PsLR76 was also observed along the first coordinate. Similarly, growth habit-based PCoA revealed three clusters with a weak grouping of accessions according to their growth habit. Cluster-I comprised 48 accessions, of which 38 were the erect and non-branching type. Four non-erect and branching and five erect and green types were also grouped in Cluster-I. Except for three erect and branching, two not identified, and one erect and non-branching, all the accessions grouped in Cluster-II were non-erect and branching. Cluster-III comprises mixed accessions from the different populations (Supplementary Figure S1). Seed color-based PCoA also revealed three clusters with a weak grouping of accessions according to their similar seed color. Accessions of different seed colors were grouped together (Supplementary Figure S2). Population structure (Q)-based PCoA also revealed three major clusters. The first cluster (Q1) was composed of 49 accessions with four breeding lines, one wild material and 44 improved cultivars, while the second (Q2) and third (Q3) clusters were composed of 84 and 132 genotypes, respectively (Figure 7). Neighbor-joining (NJ) clustering analysis, based on Nei’s standard genetic distance, was performed to assess the genetic relationship among the 265 accessions at different grouping levels. The NJ analysis of plant material-based grouping revealed a mixed clustering pattern categorizing the accessions into seven clusters. Despite a weak clustering pattern according to the type of material, the percentage of differentiation among the plant materials type groups was 5%, with the highest genetic distance observed between the WI and the other populations (GBA, CV, and BL). Cluster-I comprised 45 accessions predominantly constituted by improved cultivars (34) alongside breeding lines (8) and landraces (3). Cluster-II included 39 accessions, with 31 being improved cultivars and eight breeding lines. Cluster-III is composed almost exclusively of accessions from improved cultivars (44), except for four breeding lines. Cluster-IV (38) and Cluster-V (27) were also heterogeneous groups comprising accessions from improved cultivars, landraces, and breeding lines. Notably, Cluster-VI and Cluster-VII predominantly accommodated breeding lines (16) and landraces (12), respectively (Figure 8).

**FIGURE 9:**
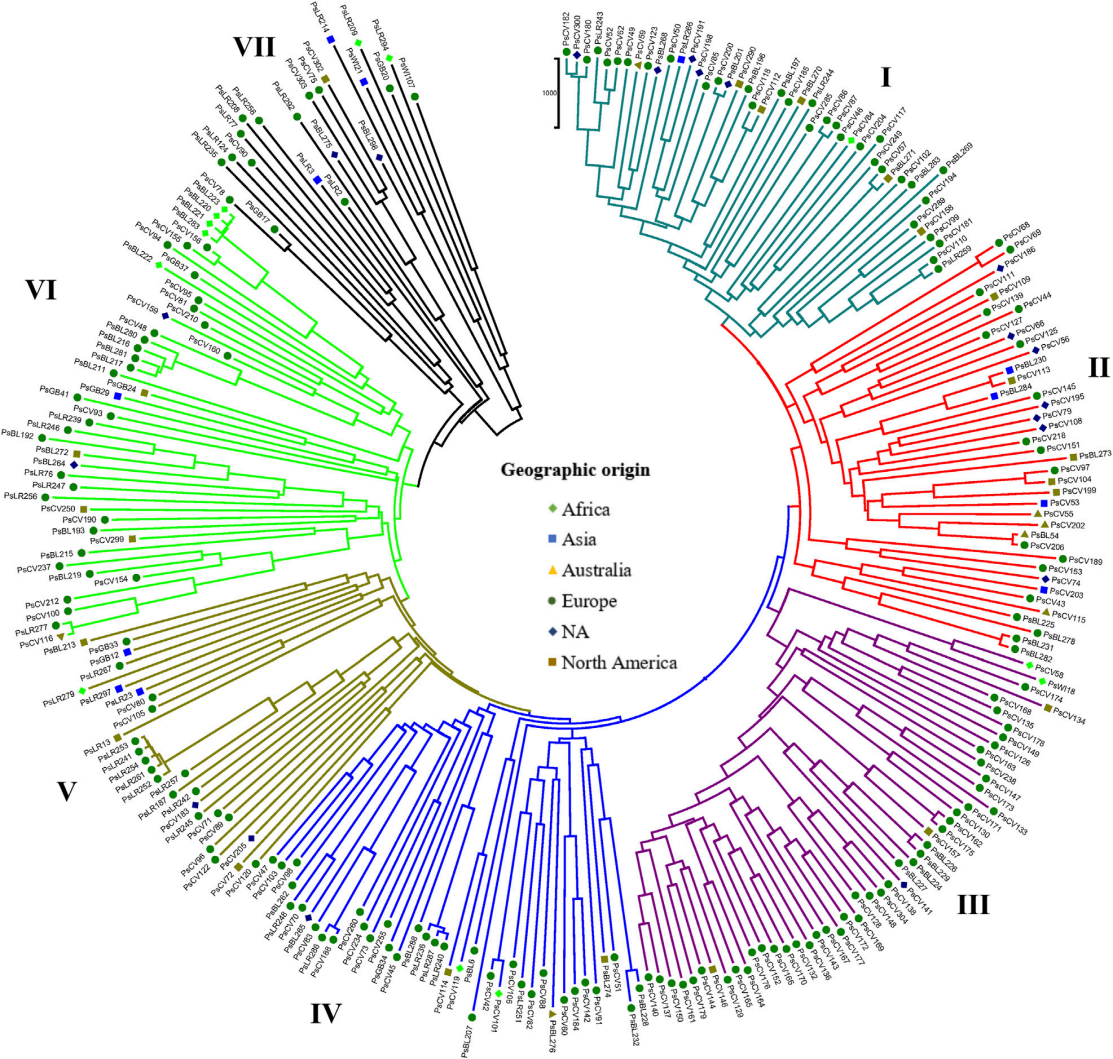
Nei’s standard genetic distance-derived neighbor-joining (NJ) clustering analysis of 265 pea accessions grouped according to their geographic origin was evaluated using the 6966 SNP markers. Symbols of the same shape and color indicate accessions of the same geographic origin.

Similarly, the NJ clustering analysis based on the geographic origin of the accessions revealed seven (I-VII) heterogeneous groups. Cluster-I comprised accessions from all geographic origins, such as 25 from Europe, 12 of unknown origin, three from Australia, two from Africa, and one from North America. Cluster-II comprised 39 accessions primarily of unknown origin (17), with additional representation of 15 from Europe, three from North America, two from Asia, one from Africa, and one from Australia. Cluster-VII included only 22 accessions, with a majority collected from Europe (9) and unknown origin (10), along with two accessions from North America and one from Asia (Figure 9).

**FIGURE 10:**
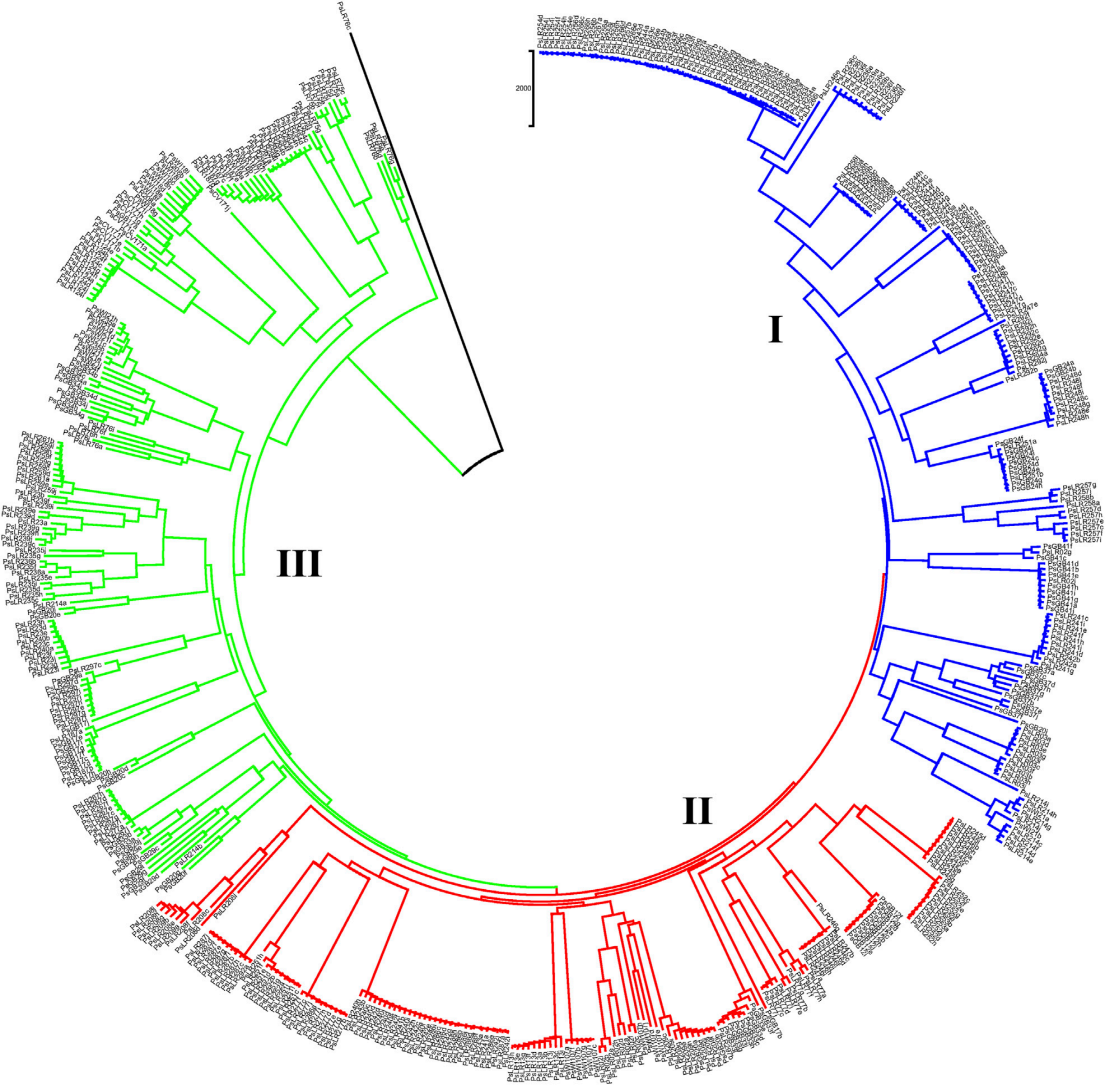
Nei’s standard genetic distance-derived neighbor-joining (NJ) clustering analysis of 54 pea populations was evaluated using the 6966 SNP markers. Symbols of the same shape and color indicate accessions of the same population.

NJ clustering analysis conducted on the 54 accessions, each represented by 10 individual samples, revealed that almost all individuals within each accession were tightly grouped together. Similar to the pattern of clustering in PCoA, NJ also confirmed the individual samples taken from each accession were genetically closely related. For instance, Cluster-I was composed of 131 individual samples, with most grouped according to their accession, except that individual samples of the PsLR243, PsLR254, PsLR256, PsLR266, and PsLR267 accessions were grouped together in the same cluster, indicating close genetic composition. Similarly, Cluster-II is composed of 161 individual samples, with most grouped according to their accession members. Finally, Cluster-III is also composed of 185 samples representing 20 accessions overall, and except for a few individuals of the accessions, each sub-cluster is composed of homogenous materials (Figure 10).

**FIGURE 11:**
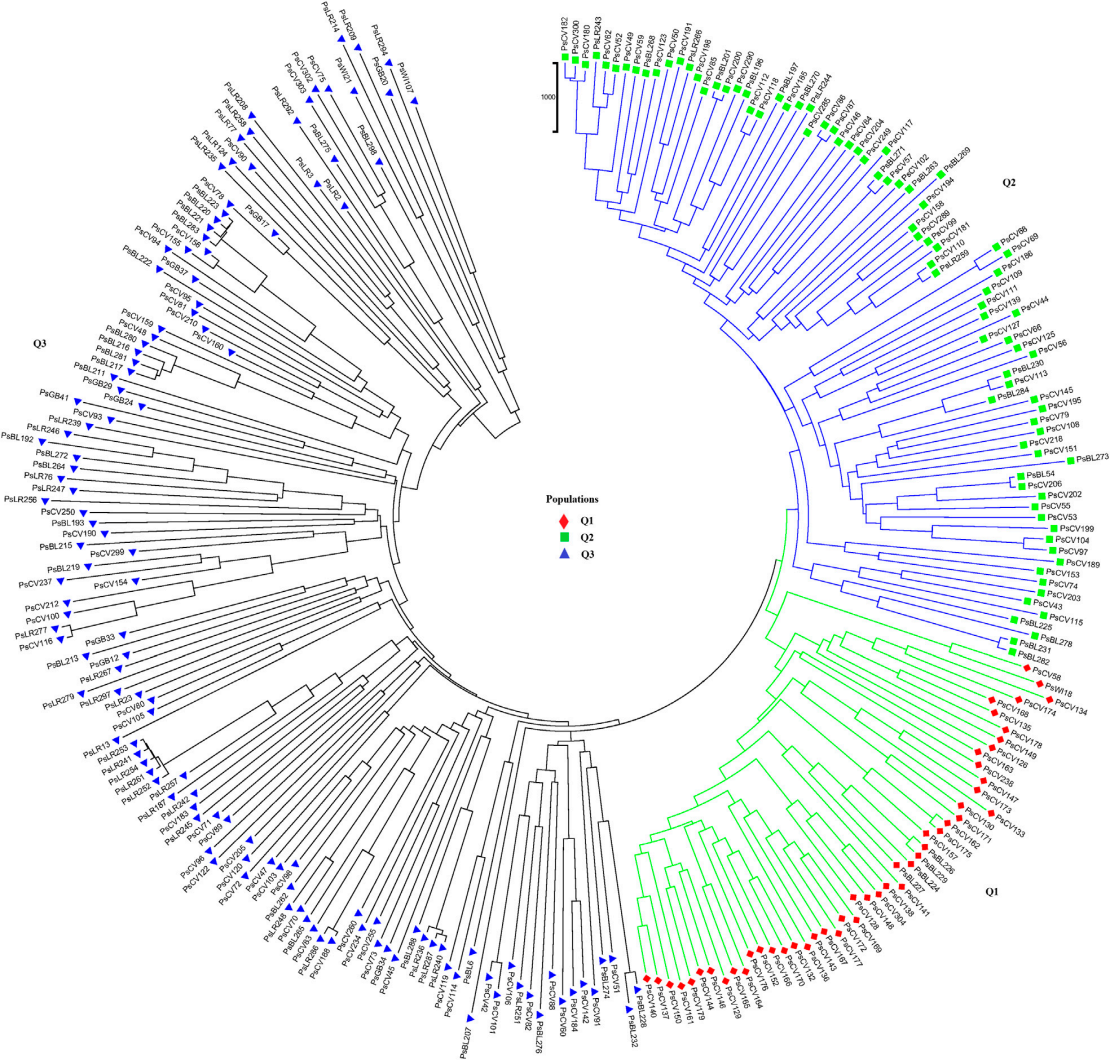
Nei’s standard genetic distance-derived neighbor-joining (NJ) clustering analysis of the 265 pea accessions grouped according to their population genetic structure generated using 6966 SNP markers. Symbols of the same shape and color indicate accessions of the same population genetic group.

Population structure (Q)-derived NJ was performed and revealed three clusters (Figure 11). Cluster-Q1 comprises 47 accessions with four breeding lines, one wild type and 44 improved cultivars. The second cluster Q2 composed of 84 total genotypes among which 16 were breeding lines, 64 improved cultivars, and four landraces. Among the total 265 genotypes, 132 (50%) of the genotypes were grouped in the third Q3 clusters. From the 132 genotypes grouped in the third cluster, 28 were breeding lines, 56 improved cultivars, nine gene bank accessions, 37 landraces, and two wild type materials.

### 3.6 Population structure and linkage disequilibrium analysis

**FIGURE 12:**
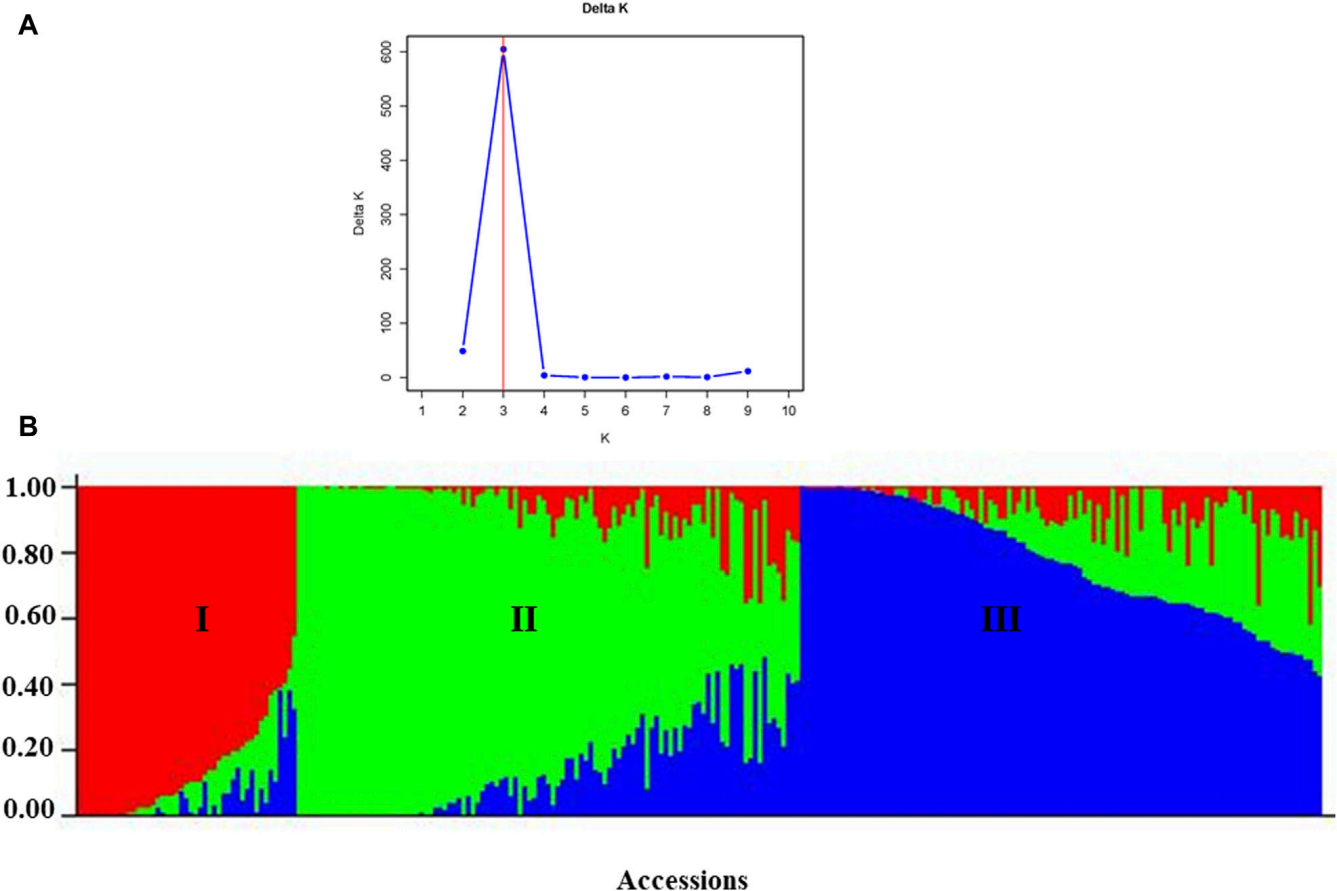
Graphic representation of the genetic structure of 265 pea accessions at **(A)** K = 3, forming three clusters and **(B)** individual accessions organized according to their membership levels in the three clusters.

According to the admixture model-based population genetic structure analysis conducted using the 6966 SNP markers, three genetic populations (K = 3) best represent the 265 pea accessions (Figures 12A,B; Supplementary Table S4). The genetic structure analysis also revealed that the first genetic group comprised 49 genotypes of improved cultivars (44), one wild type and breeding lines (4). The second genetic group is composed of 84 genotypes representing breeding lines (16), improved cultivars (64), and landrace accessions (4),(2). Finally, the third genetic group is composed of 132 heterogeneous genotypes representing breeding lines (28), improved cultivars (56), gene bank accessions (9), landrace accessions (37), and wild materials (2). The list of accessions with their respective group population structure is provided in Supplementary Table S4.

**FIGURE 13:**
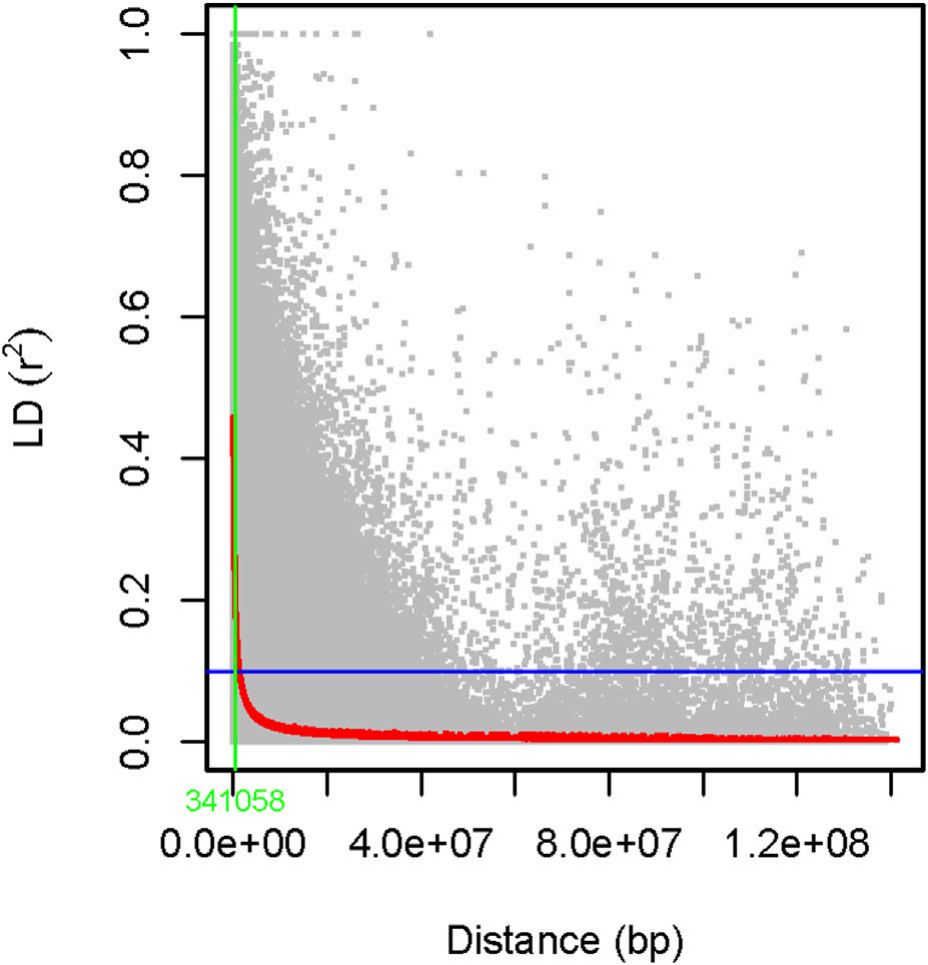
A scatter plot of genome-wide linkage disequilibrium (LD) decay as determined by r^2^ values of the marker pairs. The smoothing spline regression model fitted to LD decay is shown with a red curve line. The horizontal blue line shows the genome’s half-decay r^2^ value (r^2^ = 0.1), while the vertical green line shows the genetic distance between markers (34 Kb) at the intersection of the half-decay line and the LD decay curve.

The genome-wide LD was estimated using 6,966 filtered SNP markers located across the whole pea chromosomes, forming 344,875 SNP pairs. About 5,931 (1.7%) of the SNP pairs had r^2^ > 0.46, while 287,092 (83.2%) were in LD (r^2^ ≤ 0.1, *p* < 0.05). The LD begins to decay at r^2^ = 0.46 and drops to its half-decay at r^2^ = 0.1 at a distance of 34 kb between marker pairs (Figure 13).

## 4 Discussion

### 4.1 The germplasm collection and marker characteristics

The pea accessions utilized in this study were collected to represent a diverse set of genetic resources originating from various regions worldwide. The collection has a broad representation from Africa, Asia, Australia, Europe, and North America, and a small number of accessions have an unknown geographical origin. Additionally, the collection comprises different types of plant materials, including breeding lines, improved cultivars, landraces, unspecified gene bank accessions, and wild materials collected from seed suppliers, breeders, and gene banks. Furthermore, the accessions exhibit variability in growth habit and seed color, adding to the complexity and richness of the genetic resources in this investigation.

We developed and employed SNP and silico markers to assess the genetic diversity within the collection. Thus, for this purpose, 6966 SNP and 8,458 silico markers were carefully curated, selected, and used to analyze the germplasm collection. Both marker types demonstrated a high call rate across a significant proportion of the markers, presenting a diverse spectrum of allele frequencies ranging from rare to highly prevalent alleles and PIC values. Notably, the diversity captured by these markers surpassed that of SNP markers reported in previous studies on pea breeding lines (5,767 SNPs) (Alemu et al., 2022), pea wild-type collections (3,483 SNPs) (Barilli et al., 2018), and also in recombinant inbred lines of pea (6,540) (Aznar-Fernández et al., 2020). The reason for the higher diversity in this study may stem from the inclusion of a wide array of genotypes representing diverse ecological conditions and material types. In contrast, the number of markers obtained in this study was notably lower than in a previous study where a pea core collection of 325 accessions was used, which resulted in the identification of 11,511 SNPs and 19,514 silico markers (Rispail et al., 2023). In the study by Rispail et al., several *Pisum* species and subspecies were included in the panel, which probably led to the identification of a broader range of genetic variants and, thus, a greater number of markers.

Overall, the selected markers were shown to have good coverage across the pea chromosomes, with Chr5 contributing the highest and Chr1 the least number of markers in both the SNP and silico datasets. This consistent SNP density pattern, which was also reported in previous studies on the crop (Alemu et al., 2022; Rispail et al., 2023), suggests a widespread distribution of genetic variation in pea populations. Such wide distribution makes these SNPs ideal candidates for population genetic structure analysis and association studies, such as the construction of high-density linkage maps (Gali et al., 2018) and the identification of markers closely linked to traits (Ott et al., 2015). The distribution of markers across the seven chromosomes indicates relatively uniform coverage, albeit variations in marker density are apparent. Analyzing allele frequency distribution and polymorphic information content provided valuable insights into the genetic variation captured by the markers, with implications for further genetic analyses.

The average polymorphism information content (PIC) value of the markers was 0.26, indicating a medium level of informativeness according to the Hildebrand et al. (1992) classification. Compared to previous reports, the average PIC value (0.26) discovered across the diverse geographic origins of pea accessions in this study aligns with the expected range. More than half of the SNP markers (54.5%) have a PIC value above the average, making them highly informative and suggesting that they can detect the genetic variation among the genotypes. Comparisons with previous studies employing DArT markers reported an average PIC value of 0.29 Rispail et al., 2023) across different pea collections. In contrast, earlier studies using expressed sequence tags and genomic microsatellite (SSR) markers reported lower average PIC values (Gong et al., 2010; Rana et al., 2017). The PIC values of SNP markers are generally lower than those of SSR markers, as the average number of alleles per locus is higher in SSRs than in bi-allelic SNPs (Chao et al., 2009).

### 4.2 Genetic diversity of the pea collections

Understanding genetic diversity and population structure is crucial for designing effective breeding strategies for cultivar improvement and assembling association mapping populations of the crop. In this study, SNP and silico markers were used to evaluate the level of genetic diversity and allele distribution across 265 pea accessions representing diverse phenotypic characters and wide geographic origins. The analysis revealed comparable levels of genetic diversity among populations based on plant material type, with landraces exhibiting a slightly higher diversity. Similar magnitudes of genetic diversity across populations suggest the absence of significant diversity erosion within pea gene pools associated with specific traits of interest. Moreover, the level of genetic diversity observed in the breeding lines and improved cultivars closely resembled those of the landraces and unspecified gene bank accessions, indicating the potential for further improvement through selective breeding within the existing genetic pool. However, it is worth noting that the larger number of improved cultivars might have exerted a more pronounced influence on the estimated genetic diversity when compared to breeding lines. The same is true for considering the group of wild material, represented by only three accessions. The larger group’s genetic diversity may have a stronger impact on the overall genetic distance estimation, potentially overshadowing the contribution of the smaller group. Thus, while the analysis performed within this study has given insights into the overall genetic variation of the germplasm collection, it is essential to recognize the potential influence of sample size discrepancies. Further investigations with balanced sample sizes are needed to validate these findings and provide a more comprehensive understanding of genetic diversity across different pea populations.

Among the six populations grouped by geographic origin, accessions collected from Europe showed slightly higher genetic diversity than those from other regions. This could be due to the sample size effect, as accessions from Europe accounted for more than two-third of the total accessions, as well as the presence of unique alleles (n = 22). However, Europe might be considered a hotspot area for pea genetic diversity, making it a prime candidate for *in-situ* conservation and breeding of the crop. Overall, the relatively consistent levels of genetic diversity across the geographic origin populations (although slightly higher in European accessions) indicate maintained genetic diversity within pea gene pools. The identification of population-specific diversity patterns from the geographic origin-based analyses further underscores the importance of both geographical and genetic factors in shaping genetic diversity within pea populations. Interestingly, the similarities in diversity estimations between populations obtained using both SNP markers and silico markers confirmed that both can be used to read whole-genome sequences, identify highly informative markers, and facilitate genomic prediction and genome-wide association studies. The choice between them should align with the specific research objectives and the desired characteristics for genetic analysis within the studied population.

Based on the level of diversity within each accession, appropriate conservation and regeneration strategies should be followed to conserve the genetic integrity and diversity of pea landraces. The appropriate population size needs to be ensured, while regeneration for capturing the rare alleles with small size may lead to genetic drift, which results in the loss of some rare alleles (Allan et al., 2020). Crossa (1989) suggested that in an ideal system, a 130–200 seed sample size is required for effective regeneration and retaining rare alleles, while 30 individuals are required for completely random mating populations (FAO, 2014). However, in peas, no previously published findings were reported to determine the optimum number of seeds for conservation and regeneration. Therefore, in this study, we estimated the minimum seed sample size from each accession to capture 95% of the SNP alleles spread throughout the pea genome. The sample size required and obtained in this study (256–668) to conserve the genetic integrity of germplasm at 95% probability was higher than previous findings in sorghum (47–101) and pigeon pea (77–89). This could be mainly due to the minimum number of seeds required to conserve the genetic integrity depending on the frequency of the least common alleles or genotypes (Crossa, 1989).

### 4.3 Genetic relationships, population genetic structure, and linkage disequilibrium

Analysis of molecular variance (AMOVA) revealed significant differentiation among and within the type of plant material, geographic region of origin, growth habit, and seed color (*p* < 0.01). While the level of differentiation among geographic origin groups was low (3%), a more pronounced differentiation was observed among the type of plant material (5%), seed color (7%), and growth habit (7%). This indicates that factors such as seed color and growth habit contribute more significantly to genetic differentiation than geographical origin, which is also in line with other studies (Keneni et al., 2005; Glaszmann et al., 2010). Therefore, solely relying on standard passport data such as geographic origin and plant material type as an index of genetic diversity will not capture the full extent of variation within a crop species. While geographic diversity undeniably contributes significantly to the overall genetic diversity essential for crop improvement, our findings suggest that prioritizing morphological traits such as seed color and growth habit over plant material type and geographic origin could yield substantial benefits. Other studies have shown that selecting accessions based on phenological traits and including different ecotypes will additionally enrich the genetic diversity of a panel (Smýkal et al., 2015). This underscores the critical need for comprehensive characterization of gene bank materials across a diverse spectrum of traits and emphasizes the importance of expanding the information included in passport data as well as making the data available in public databases. By focusing on traits that are directly linked to agronomic performance and end-user preferences, breeders can make more informed decisions in selecting germplasm for breeding programs, thereby enhancing the efficiency and effectiveness of crop improvement efforts.

It is possible that the close relationship between accessions from different plant materials collected from different geographic origins could be a consequence of gene flow (allele distributions) through different seed exchange channels, resulting in low differentiation. Ten individuals were also considered from each accession as a separate population to deep scan the variation of each accession, and this revealed 90% within-accession variation and 10% differentiation among accessions. Thus, there was high genetic differentiation among the 54 accessions represented by 10 individuals. Within each accession (represented by 10 individuals), there is considerable genetic diversity, which implies that each accession likely contains multiple genetic variants, contributing to the overall genetic diversity within that particular accession. When the analysis was repeated using only one randomly selected individual per accession (totaling 54 individuals), the distribution of genetic variation changed significantly. In this case, most of the genetic variation (97%) was observed within populations, while a much smaller proportion (3%) was observed among populations. This supports that the choice of the sampling strategy (i.e., selecting multiple individuals per accession *versus* selecting only one individual per accession) will have a substantial impact on the observed genetic variation of these heterogeneous material types.

The clustering and PCoA analysis performed in this study provided insights into the genetic relationships among the pea accessions. The results showed low differentiation between groups, suggesting a strong gene flow among them. PCA based on plant material types divided all the 265 pea accessions into three main groups, with Cluster-I and Cluster-III dominated by breeding lines and improved cultivars, while Cluster-II had a more heterogeneous composition (Figure 4). The clustering pattern of breeding lines and improved cultivars was further supported by the short pairwise genetic distance between the two populations. This observation aligns with previous studies employing molecular markers. For instance, Rispali et al. (2023) conducted PCoA on 325 using DArT markers, resulting in the identification of three clusters, and, similarly, Liu et al. (2022) investigated 323 diverse accessions using SSR markers and reported clustering patterns comparable to those observed in our study. A closer examination of the accessions within these clusters reveals interesting associations. Cluster-I predominantly includes garden pea cultivars of snow pea and snap pea types, suggesting that there is a strong association between the genetic characteristics of these accessions and the traits typically found in garden peas, Cluster-II is primarily characterized by Nordic heirloom cultivars (old landraces), and nearly all accessions with brown and orange seed coat testa (30 out of 33) are found in this cluster, which is a common genetic trait among these landraces. Meanwhile, Cluster-III is mainly constituted by modern cultivars of yellow dry peas utilized for cooking or fodder, with 39 of 49 accessions displaying yellow or cream-colored seed coats. Understanding the genetic associations with specific traits, as observed in these clusters, can inform breeding programs aimed at developing cultivars with desired characteristics.

The landrace gene bank accessions and wild materials were deep scanned (10 individuals per accession) to observe their genetic distance. Clustering analysis revealed that individual samples taken from each accession were grouped tightly, which indicates similar genetic composition. Whereas the wide variation observed across the landrace accessions agreed with previously reported findings on 120 pea landrace accessions using inter-simple sequence repeats (ISSR) markers (Lázaro and Aguinagalde, 2006; Arif et al., 2024). However, an outlier accession (PsLR76c) was observed in both the PCoA and NJ. An anomaly occurred during the sowing of this specific accession, as it was discovered that peas from a neighboring plot accidentally became trapped in the sowing machine, potentially leading to cross-contamination with the wrong plot. Thus, the outlier observed in this accession is most likely attributed to a mix-up from the adjacent plot during the sowing process. Unexpectedly, the accessions PsLR243, PsLR254, PsLR256, PsLR266, and PsLR267 were placed in the same cluster. PsLR243, PSLR254, and PSLR256 are known Swedish landraces that have been grown and preserved by farmers for many generations (Aloisi et al., 2022), while PsLR266 and PsLR267 originated from Asia but were once part of the pea breeding program of the old seed company Weibull, today owned by the Swedish agricultural cooperative Lantmännen.

Clustering and PCoA analysis showed a lack of a clear relationship between pea accessions and their geographical origin, as evidenced by overlapping clusters. This aligns with previously published research findings on peas using simple sequence repeat (SSR) markers (Keneni et al., 2005; Solberg et al., 2015), indicating the occurrence of overlapping genotypes from different agro-ecological regions. Within each cluster, specific accessions exhibited unique genetic profiles, suggesting potential genetic distinctiveness or admixture within these subgroups. The observed displacements of specific accessions in each cluster highlight the complexity of the genetic relationships among the pea accessions, with factors beyond geographic origin contributing to the observed genetic variation. This can probably be attributed to the complex interplay of historical, cultural, biological, and environmental factors, such as seed exchanges among breeders and farmers or intentional transportation to different regions for cultivation. Interestingly, most of the unspecified gene bank accessions were grouped closely with genotypes of European origin, indicating a probable European provenance for these accessions.

Identification of LD between markers is highly useful because it is a prerequisite before conducting any association studies (Meng et al., 2003). In this study, we determined that about 83.2% of the SNP marker pairs had significant LD (r^2^ > 0.1, *p* < 0.05). This agreed with previously published findings on peas (Alemu et al., 2022). The scale at which LD decays is one of the main factors to consider when evaluating the density of markers necessary to achieve sufficient power in association mapping or genomic selection approaches. This is particularly important in species such as peas, which have a very large genome (∼4.45 Gb). Moreover, our germplasm collection was dominated by cultivars that have undergone selective breeding aimed at fixing favorable alleles. However, these cultivars may also retain genetic diversity from ancestral populations or breeding efforts introducing novel alleles. This interplay between selection and genetic diversity contributes to the variable LD patterns observed across the genome. Understanding these dynamics informs marker selection strategies and enhances the effectiveness of genetic analyses in cultivar-dominated populations.

The identification of three distinct genetic populations (K = 3) in the population structure analysis suggests a clear separation within the pea germplasm, indicating potential avenues for targeted breeding efforts. The observed heterogeneity among gene bank accessions and landraces implies a rich diversity within these groups, which can be leveraged to broaden the genetic base in breeding programs. This result was comparable to those of previous studies where STRUCTURE analysis also grouped pea collections into three genetic populations (Rana et al., 2017, Siol et al., 2017) but contrasts with research on peas that reported two genetic groups (Alemu et al., 2022; Liu et al., 2022). When breeding new cultivars, focusing on accessing and incorporating genetic material from all identified populations could lead to improved traits and increased genetic diversity. Additionally, understanding the specific genetic characteristics associated with each population allows breeders to tailor their selection criteria for desired traits. In terms of conservation, recognizing the unique genetic composition of landraces, gene bank accessions, and wild materials again underscores the importance of preserving these diverse genetic resources.

## 5 Future research directions

Future studies could explore several paths to build upon the findings of this study and further enhance our understanding of pea genetics and breeding. Integrating genomic data with phenotypic information through multi-omics approaches can unravel complex trait networks and facilitate the identification of candidate genes for targeted breeding efforts (Gali et al., 2019). Moreover, expanding the scope of germplasm exploration to untapped regions and wild relatives of peas could uncover novel genetic variations for broadening the genetic base (Yang et al., 2021). Overall, future research efforts should aim to translate genomic insights into clear outcomes for pea breeding and conservation, thereby contributing to the sustainability and resilience of pea production in the face of future challenges.

Understanding diversity in peas provides a genetic “insurance policy” that can be tapped into for breeding new cultivars with improved traits, such as resistance to pests and diseases, tolerance to environmental stresses, and enhanced nutritional quality. By preserving and utilizing this genetic variation, breeders can develop cultivars that are better adapted to changing environmental conditions, evolving pest and disease pressures, and shifting market demands. Additionally, the availability of diverse genetic resources facilitates the exploration of novel traits and the development of more resilient and productive cropping systems.

## 6 Conclusion

This study reveals the rich genetic diversity present within the pea collection using SNP and silico markers to explain the genetic relationships among the accessions. The results highlight the importance of factors such as plant material type, seed color, and growth habit in shaping genetic diversity. The identification of distinct genetic populations provides valuable insights for targeted breeding efforts and conservation initiatives. Thus, this study contributes to the much-needed ongoing efforts to characterize landraces and diverse germplasm, addressing the challenge of their genetic heterogeneity and unlocking previously unexplored genetic diversity. Moving forward, integrating genomic data with multi-omics approaches and expanding germplasm exploration to untapped regions and wild relatives of pea offer promising avenues for further research. By leveraging genetic diversity, pea breeding programs can be enhanced and contribute to the sustainability and resilience of plant protein production in the face of future challenges. Information from this study also supports the pea gene bank professionals in sampling strategies for conservation and regeneration to maintain genetic integrity and variability.

## Data Availability

The data is publically available with its accession number PRJNA1071600. Here is the link: https://www.ncbi.nlm.nih.gov/bioproject/PRJNA1071600.

## Supplementary material

SUPPLEMENTARY TABLE 1 List of pea (Pisum sativum) accessions used in the present study with their passport data (accession code, name of the accession, accession number, source of accessions), geographic origin, plant material type, seed color, and growth type.

SUPPLEMENTARY TABLE 2 Description of the accessions, plant material type, number of loci with private alleles, and list of loci with private alleles in pisum sativum accessions evaluated using Diversity Arrays Technology sequencing (DArTseq) method.

SUPPLEMENTARY TABLE 3 Estimated seed sample sizes required for regenaratoion and conservation to capture 90%, 95%, and 99% of the alleles with an expected probability of 90%, 95% and 99% using the least DArTSeq–SNP allelic frequency of the landrace, gene bank and wild material accessions of pisum sativum.

SUPPLEMENTARY TABLE 4 List of pea genotypes with thier memebership population genetic groups. Q1 = Population genetic group 1, Q2 = Population genetic group 2, and Q3 = Population genetic group 3.

SUPPLEMENTARY FIGURE 1 Bi-plot of principal coordinate analysis (PCoA) depicting the genetic relationship of the 265 pea accessions grouped based on their seed color generated using 6966 SNP markers. A symbol of the same shape and color corresponds to accessions of the same population genetic group.

SUPPLEMENTARY FIGURE 2 Nei's standard genetic distance neighbor-joining (NJ) clustering analysis of 265 pea accessions grouped based on seed color was generated using 6966 SNP markers. Accessions sharing a symbol with the same shape and color belong to the same plant material type.
