# Supplementary figures and images for: Genetic diversity and population structure analysis of a diverse panel of pea (Pisum sativum)
Source: Front Genet. 2024 May 30;15:1396888. doi: 10.3389/fgene.2024.1396888 (PMC11169732; doi:10.3389/fgene.2024.1396888)

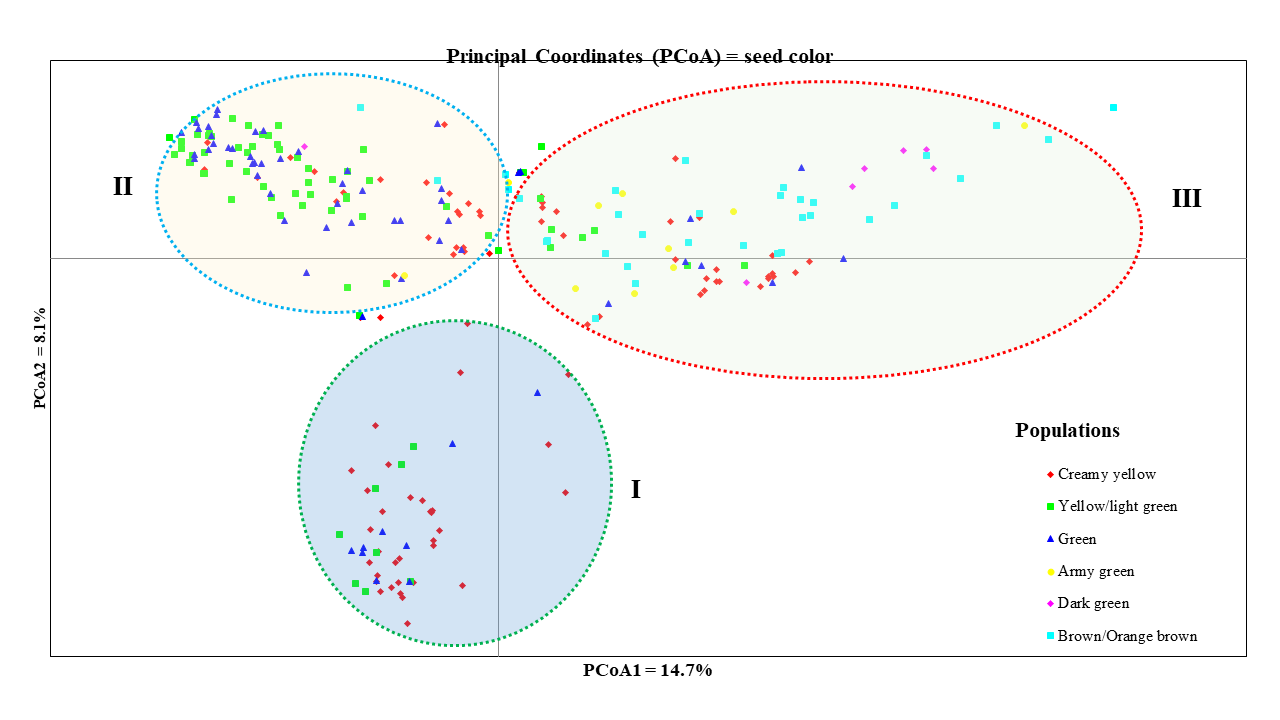

Supplement: Supplementary file 3 [file Image2.TIF]

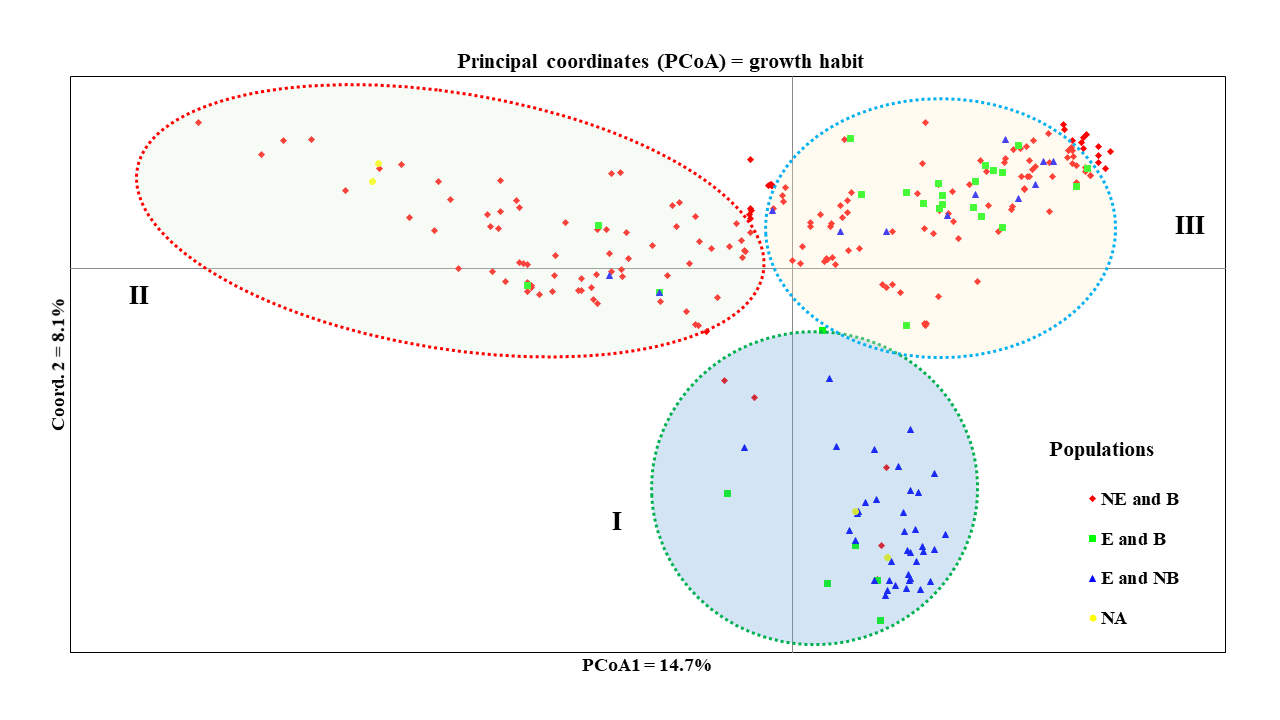

Supplement: Supplementary file 4 [file Image1.TIF]
